# Genome-wide analysis of polygalacturonase gene family from pear genome and identification of the member involved in pear softening

**DOI:** 10.1186/s12870-019-2168-1

**Published:** 2019-12-27

**Authors:** Suling Zhang, Min Ma, Huping Zhang, Shaoling Zhang, Ming Qian, Zhen Zhang, Weiqi Luo, Jinbu Fan, Zhiqiang Liu, Libin Wang

**Affiliations:** 1 Centre of Pear Engineering Technology Research, State Key Laboratory of Crop Genetics and Germplasm Enhancement, Nanjing Agricultural University, Nanjing, 210095 Jiangsu China; 2 USDA, ARS, U.S. Horticultural Research Laboratory, 2001 S. Rock Road, Fort Pierce, FL 34945 USA

**Keywords:** Polygalacturonase, Gene family, Pear fruit, Firmness, Ethylene response factor

## Abstract

**Background:**

Polygalacturonase (PG), as an important hydrolase participating in the degradation of pectin, plays an important role in softening process of fruit. However, information on *PG* gene family in pear genome and the specific member involved in fruit softening is still rudimentary.

**Results:**

In this study, a total of 61 *PG* genes, which could be divided into six subclasses, were identified from the pear genome with diverse chromosome locations, gene structures, motifs and *cis*-acting elements. Most *PbrPGs* were derived from WGD/segmental duplication blocks, and purifying selection was the main driving force for their expansion. The expression profiles of *PbrPGs* in pear were tissue/development-stage/cultivar-dependent. During ‘Housui’ pear storage, associated with the reduction of firmness was the accumulation of PG activity. Totally, 28 *PbrPGs* were expressed during fruit storage, which could be classified into five categories based on different expression patterns; most demonstrated an increased trend. Of these, *PbrPG6* were proposed to account for pear softening in combination of the phylogenetic and correlation analysis among firmness, PG activity and *PbrPGs*. By constructing the silencing vector, a higher firmness was observed in *PbrPG6*-silenced fruit when compared with that of the control (empty vector). In a further study, we found that the expression of *PbrPG6* was regulated by postharvest 1-MCP/ethrel treatment, and several *PbrERFs* might function in this process.

**Conclusions:**

We identified 61 *PbrPG* genes from pear genome; of these, *PbrPG6* was involved in fruit softening process; furthermore, the expression of *PbrPG6* might be under the control of PbrERF. This study provides a foundation for future work aimed at elucidating the molecular mechanism underlying pear softening.

## Background

Ripening & senescence of horticultural fruit is a very complex process, which accompanies with the changes in color, texture and flavor [1]. As one of the most obvious phenomena, the reduction of firmness during storage could enhance the sensitivity of fruit to mechanical damage and thus shorten their shelf life [2]. Fruit softening is mainly due to the alternation in cell wall structure and composition, including cellulose, hemicellulose, and pectin [3]. Pectin, a major component of the primary cell wall, play a critical role in cellular structural integrity and cell adhesion [4].

Belonging to one of the largest hydrolase families, polygalacturonase (PG), which was discovered half a century ago, has known to be involved in various processes of plant development, such as flower development, fruit ripening & senescence and organ abscission [5–7]. PG plays an important role in pectin disassembly, and could be divided into three types based on different catalytic processes, including endo-PGs, exo-PGs, and rhamno-PGs [6]. Until recently, *PG* family genes have been identified from various plants, such as *Arabidopsis*, *Oryza sativa*, *Brassica rapa*, *Populus*, cucumber, watermelon, tomato, mango, apple and peach [6–8]. Eleven members from *Populus* were proposed to be involved in flower development, while two related to leaf abscission under salt stress [9]. Of 54 *SlPGs* identified from tomato fruit, members in clade A and B were involved in fruit and abscission zone development, while members from clade C, D, and F in flowering development [7]. Three *PpPGs* supposedly participated in the softening process of peach [8]. These results implied that there was extensive functional differentiation among plant *PG* genes.

Ethylene plays an important role in the ripening & senescence process of climacteric fruit [10]. Mutation of an ethylene receptor, *Never-ripe* (*Nr*), suppressed the ripening process of tomato fruit; furthermore, the expression of 37% genes was altered in transgenic fruit, causing distinct seed number, ascorbate & carotenoid abundance [11]. As the final response gene in the ethylene signaling pathway, ethylene response factors (ERFs) trigger an ethylene response and regulate fruit ripening by binding to the promoters of several ripening-related genes, such as *pectin methylesterase* (*PME*), *1-aminocyclopropane-1-carboxylic acid oxidase* (*ACO*), and *PG* [10, 12, 13]. Electrophoretic mobility shift assay (EMSA) demonstrated a specific binding of *CpERF9* to the promoters of *CpPG5* and *CpPME1/2* in papaya, via the GCC-box motif [13]. A majority of ERFs would activate the transcription of ripening-related genes, while some demonstrated a reverse impact [10].

Pear, as a respiratory climacteric fruit, is popular because of its juicy and delicious taste [14, 15]. During fruit storage, associated with the change in the composition of flavor contributors was the reduction of firmness [14, 16–18], which PG might played an important role in [19]. However, our knowledge on pear *PG* gene family and the specific member involved in fruit softening was still rudimentary. In this study, the identification of *PG* genes from pear genome was performed to analyze their chromosome localizations, gene structures, motif composition as well as *cis*-acting elements. Their expression profiles in different tissues as well as during fruit development/storage were determined. In combination with the results of phylogenetic and correlation analysis among firmness, PG activity and *PbrPGs* transcripts, member playing an important role during the softening process of pear fruit was identified and functionally validated through the construction of transient silencing vector. Furthermore, the candidate ERFs possibly involved in regulating the expression of the key *PG* gene were summarized.

## Result

### Identification and phylogenetic analysis of *PbrPGs*

Totally, 61 *PG* genes were identified from pear genome, which were named as *PbrPG1–61* based on their chromosomal location (Additional file 2: Table S1). Of these, 43 genes contained the conserved domains I, II, III and IV; *PbrPG18* and *52* lacked the domain I; 14 members did not possess the domain III; *PbrPG2* and *3* lacked the domain IV; *PbrPG8* lacked the domain II and III (Additional file 1: Figure S1 and Additional file 2: Table S1). Eighty ESTs hits were identified for all *PbrPGs* with the greatest number for *PbrPG7*, *46*, *57*, and *58* (Additional file 2: Table S2).

**Fig. 1:**
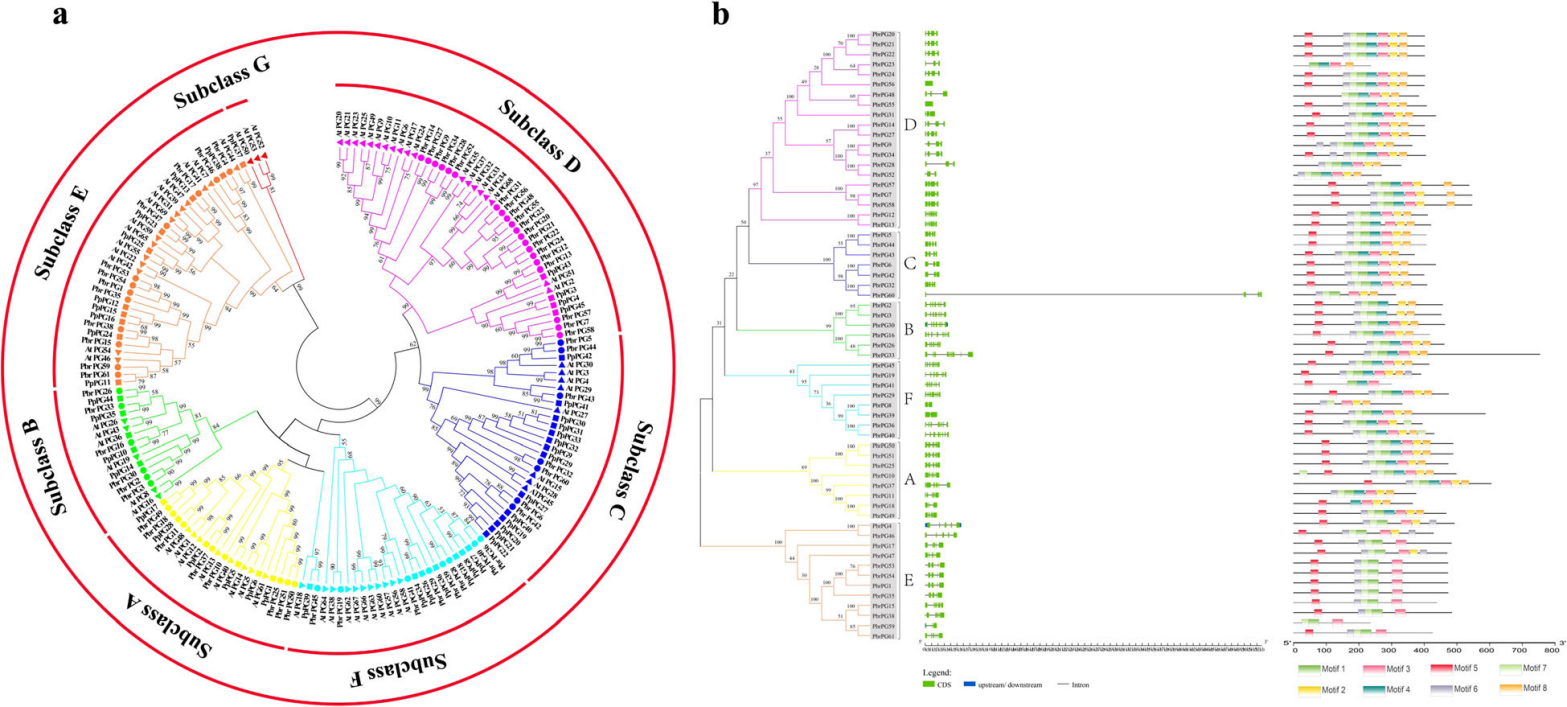
Characterization of *PG* genes from Pyrus bretschneideri and other plants. **a** Phylogenetic tree of *PGs* from plants, including Pyrus bretschneideri (*PbrPG*), *Arabidopsis thaliana* (*AtPG*), and peach (*PpPG*). Circles represented *PbrPGs*; triangles indicated *AtPGs*, and squares represented *PpPGs*. Different colors represented distinct subclasses. **b** Gene structures and conserved motifs of *PbrPGs*/PbrPGs. The left part indicated the phylogenetic tree of *PbrPGs*, branches of different colors represented different subclasses. The middle part was exon/intron structures of *PbrPGs*. Green boxes indicated the exons, blue boxes represented UTRs, while black lines represented introns. The right side showed the distribution of conserved motifs in PbrPGs. Boxes with different colors represented eight different conserved motifs

Referring to the biological classification of *PGs* from peach and *Arabidopsis*, *PbrPGs* could be grouped into six subclasses (subclasses A to F), including 8 (A), 6 (B), 7 (C), 20 (D), 12 (E) and 8 (F) members, respectively (Fig. 1a). Subclass G was composed of three *PG* genes from *Arabidopsis*, excluding any members from pear and peach (Fig. 1a). Besides, *PbrPG48*, *55*, *31*, *56*, *23*, *20*, *21*, *22*, *24* and *PbrPG9*, *34*, *28*, *52* in subclass D constituted two special subgroups without members from other species (Fig. 1a).

The length, molecular weight, isoelectric point (*pI*), extinction coefficient, instability index, aliphatic index and grand average of hydropathicity (GRAVY) of PbrPGs were within the ranges of 234–753 amino acids, 24.74–82.23 kDa, 4.61–9.70, 10,345–88,975, 23.39–61.89, 28.53–97.47, − 0.366-0.109, respectively (Additional file 2: Table S1). Instability index is used to determine whether the protein is stable in a test tube (≤40, probably stable; > 40, probably not stable) [15]. Thus, most PbrPGs were predicted to be stable (Additional file 2: Table S1). GRAVY values for most PbrPGs were below zero, suggesting that they were hydrophilic (Additional file 2: Table S1). SignalP 4.1 analysis revealed that PbrPG1–3, 5–7, 12–16, 18, 20–27, 29, 33–40, 42, 43, 45, 47, 49–51, 53–58 contained signal peptides (Additional file 2: Table S1).

### Gene and protein feature of *PbrPGs*

As shown in Fig. 1b, the number of exons (intron) in *PbrPGs* ranged from 1 (0) to 11 (10); members in subclasses A, B and F generally possessed more exons/introns than others; in addition, the exon/intron structure in the same subclass was relatively conserved. Furthermore, the average intron/exon number of *PbrPGs* were higher/larger than those in the whole genome; and the average GC3 percentage of *PbrPGs* was lower than the average level in the whole genome (Additional file 2: Table S3).

One hundred five *cis*-acting elements were identified from the promoters of *PbrPGs,* which could be divided into eight categories (Additional file 2: Table S4), including conserved promoter motifs (*n* = 2), light responsive elements (*n* = 26), phytohormone responsive elements (*n* = 12), defense/abiotic stress responsive elements (*n* = 5), tissue/organelle specific elements (*n* = 7), pathogen/elicitor/wound responsive elements (*n* = 1), miscellaneous elements (*n* = 10) and elements with unknown functions (*n* = 42) [15]. Their distribution in *PbrPGs* were distinct (Additional file 2: Table S4). Comparative analysis of upstream regions of close paralogs showed divergence in the promoters of duplicated genes (Additional file 1: Figure S2).

Eight motifs were found in PbrPGs, with diverse distributions (Additional file 2: Table S5): 96.7% members contained motif 1 and 3, while several motifs only existed in certain subclasses (for example, subclass E did not possess motif 2, 4 and 8).

### Chromosomal location, gene duplication and Ka/Ks analysis

**Fig. 2:**
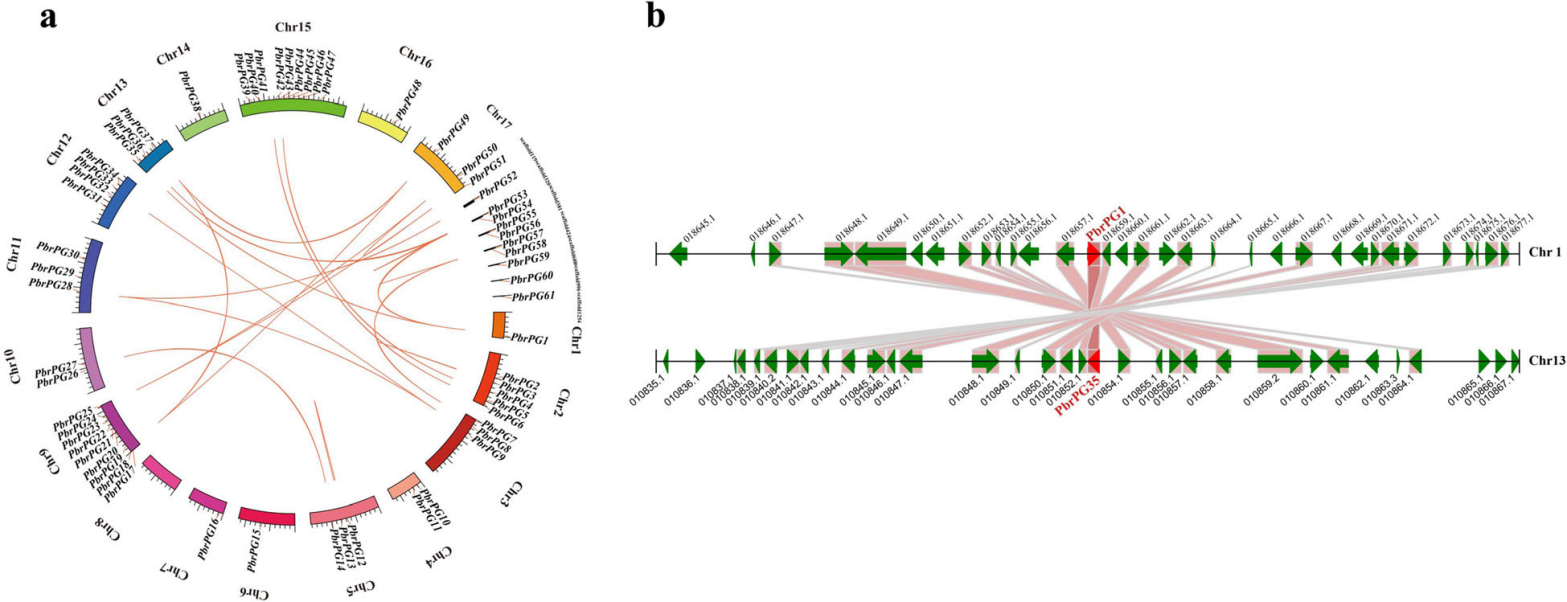
Localization and synteny of *PbrPGs* in pear genome. **a** Localization of *PbrPGs* on the chromosomes/scaffold. Chromosome or scaffold number was indicated on the outer side. Different colors represented different chromosomes. The WGD/segmental duplication genes were connected by orange lines. **b** Synstenic relationship of *PbrPG1* and *PbrPG35*, 100 kb on each side. WGD/segmental duplication gene pairs were connected with bands. The chromosome segment was indicated by black horizontal line, and the broad line with arrowhead represented gene and its transcriptional orientation. The text besides the gene was the gene locus identifier suffix. *PbrPGs* were shown in red, while other genes in black

Most *PbrPGs* were distributed on 16 chromosomes with an uneven distribution (Fig. 2a). Twenty-six genes were derived from WGD/segmental duplication; 22 and 12 members were assigned to dispersed and tandem duplication block, respectively; on the other hand, only 1.64% was derived from proximal duplication (Additional file 2: Table S6). An all-vs.-all local BLASTP based on a method similar to the one used for PGDD was performed across the whole pear genome to identify synteny blocks. Conserved synteny was observed in the regions containing *PbrPGs*. Take *PbrPG1* & *PbrPG35*, highly conserved synteny was observed in the regions containing these genes (Fig. 2b).

As shown in Additional file 2: Table S7, seven duplicated gene pairs have similar Ks values (0.159–0.237), suggesting that they might be derived from the recent WGD/segmental duplication (30–45 MYA); 11 gene pairs had smaller Ks values (0–0.141), suggesting that they may come from more recent WGD/segmental duplication; *PbrPG5* & *PbrPG42* (Ks ~ 2.000) might arise from the γ triplication (~ 140 MYA) [20]. Moreover, the Ka/Ks ratios of 15 paralogous gene pairs were less than one, implying that purifying selection was the primary driving force for *PbrPGs* [15].

### Expression profiles of *PbrPGs* in different tissues and during pear fruit development

**Fig. 3:**
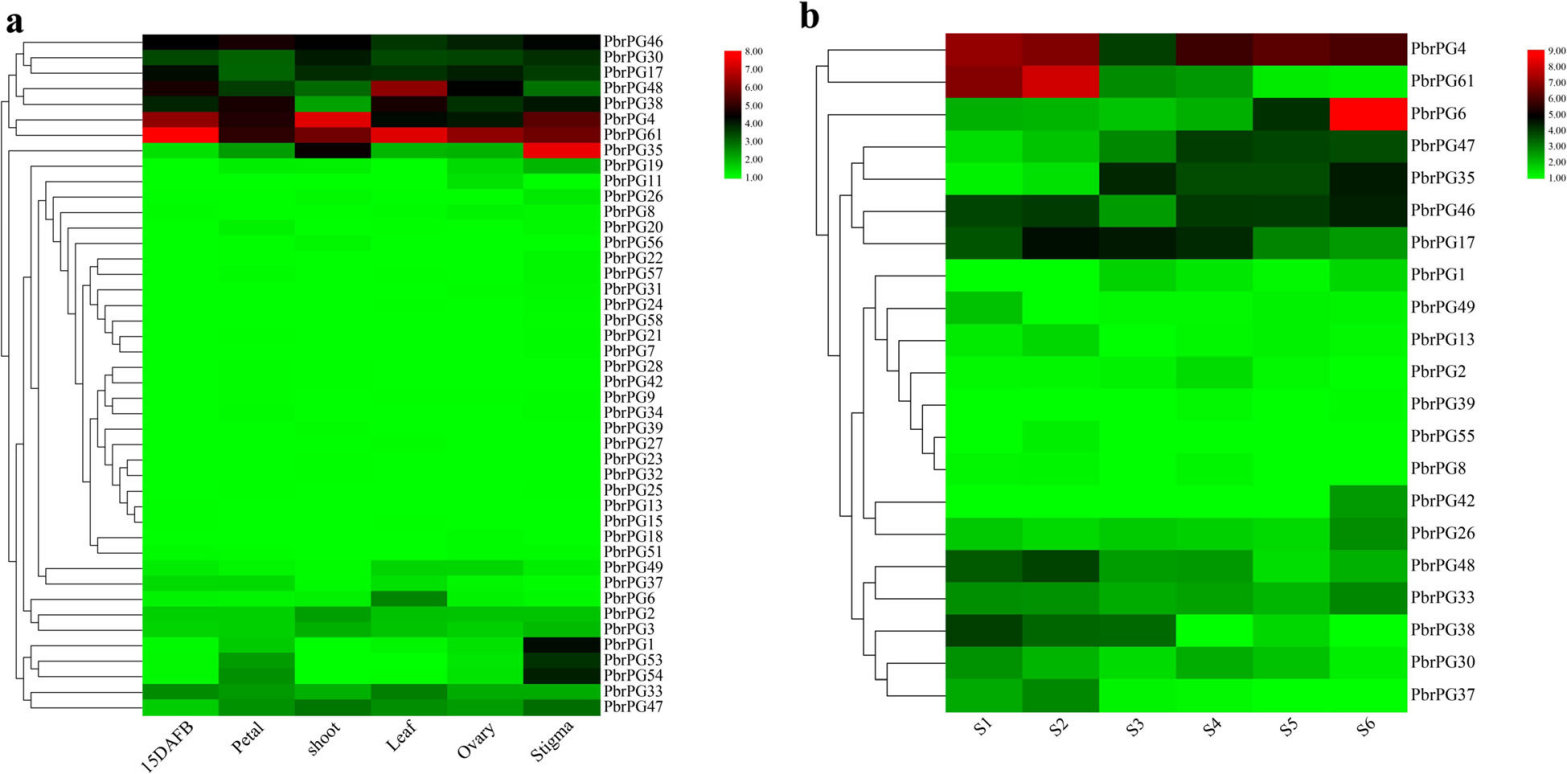
Expression profiles of *PbrPGs* in different tissues and during fruit development of pear. **a** Expression profiles of *PbrPGs* in different tissues, including 15 DAFB (15 days after full bloom), petal, shoot, leaf, ovary, stigma. Data represented the mean FPKM value of three biological replicates. **b** Expression profiles of *PbrPGs* during fruit development, including fruit-setting stage (S1), physiological fruit dropping stage (S2), fruit rapid enlargement stage (S3), a month after fruit enlargement stage (S4), pre-mature stage (S5) and mature stage (S6). Data adapted from Zhang et al. (2015). Color scale at the top represented log2 transformed (FPKM + 1). Green indicated low level, black indicated a medium level, and red indicated high level

The expression profiles of *PbrPGs* in different tissues of ‘Yali’ pear were distinct, with the highest total abundance in stigma and lowest in petal. 34, 24, 26, 25, 32 and 23 genes were expressed in stigma, shoot, ovary, leaf, petal and 15 DAFB fruit, respectively; and the abundance of *PbrPG61* mRNAs was highest in fruit, petal, leaf and ovary, while *PbrPG4* and *PbrPG35* demonstrated the highest expression in shoot and stigma, respectively (Fig. 3a).

Twenty-one *PbrPGs* were transcribed during ‘Housui’ fruit development with diverse expression patterns: *PbrPG6*, *35* and *42* mRNAs showed an increased trend throughout fruit maturity, while *PbrPG37* and *38* decreased; the expression of *PbrPG4*, *33* and *46* was down-regulated during early stage before an increase, while a opposite phenomenon was observed for *PbrPG2*, *13*, *17*, *37*, *47*, *49*, *55* and *61*; on the other hand, the transcript abundance of other members fluctuated (Fig. 3b) [21]. Moreover, the expression profiles of *PbrPGs* during fruit development were cultivar-dependent (Additional file 1: Figure S3) [21].

### Identification of *PbrPGs* involved in pear softening

**Fig. 4:**
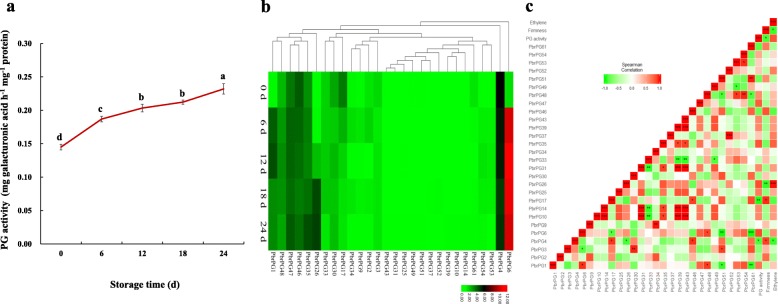
Dynamic changes of PG activity and *PbrPG* mRNAs during ‘Housui’ pear storage. **a** Dynamic change of PG activity. Data represented the mean value ± SE of three biological replicates. Different small letters with the same treatment mean significant difference among samples (*p* < 0.05). **b** Heatmap of the expression profiles of *PbrPGs*. Uniform and defect-free ‘Housui’ pear fruit were selected, randomly divided into several groups, packed with plastic bags, and then stored at 25 °C. Samples were taken every 6 d until decay rate over 20%. Color scale at the top represented log2 transformed (FPKM + 1). Green indicated low level, black indicated a medium level, and red indicated high level. **c** Correlation coefficients among firmness, ethylene evolution, PG activity and *PbrPG* mRNAs. Pearson correlations between attributes were visualized as a heat map, where negative correlations are represented in green and positive correlations in red

During ‘Housui’ pear storage, a^*^ value in pericarp, weight loss and decay rate steadily accumulated, while firmness in fruit cortex decreased, which was associated with the alternation of ethylene evolution and respiration rate—both increased from 0th d to 18th d, and then decreased (Additional file 1: Figure S4 and Table 1). For PG activity, it accumulated throughout storage, with a 52% increment (Fig. 4a).

**Table 1:** Quality changes during ‘Housui’ pear storage ^a^

Attribute		Storage time/d				
		0	6	12	18	24
Weight loss (%)		0.00 ± 0.00 ^b^	0.72 ± 0.19	1.45 ± 0.21	2.35 ± 0.39	3.10 ± 0.46
Firmness (N)		254.00 ± 12.17	223.67 ± 6.66	131.33 ± 21.46	107.33 ± 4.93	95.33 ± 3.06
Decay rate (%)		0.00 ± 0.00	0.00 ± 0.00	9.00 ± 1.00	17.00 ± 1.00	22.00 ± 2.00
Color (Pericarp)	L*	55.41 ± 0.34	54.19 ± 1.79	55.42 ± 0.55	54.73 ± 1.30	55.85 ± 2.58
	a*	1.98 ± 0.21	2.22 ± 0.58	5.05 ± 1.56	8.80 ± 1.12	9.68 ± 1.56
	b*	31.97 ± 0.35	34.00 ± 1.89	33.80 ± 0.74	34.25 ± 0.82	34.94 ± 3.28
Color (Cortex)	L*	66.24 ± 2.45	58.73 ± 2.97	61.23 ± 2.64	60.05 ± 3.19	66.00 ± 5.60
	a*	−1.46 ± 0.11	−1.52 ± 0.13	−1.51 ± 0.19	−1.64 ± 0.14	−1.53 ± 0.09
	b*	8.08 ± 0.34	6.89 ± 0.49	7.96 ± 0.70	8.20 ± 0.43	7.86 ± 0.99

^a^ Uniform and defect-free ‘Housui’ pear fruit were selected, randomly divided into several groups, packed with plastic bags, and then stored at 25 °C. Samples were taken every 6 d until decay rate over 20%

^b^ Data represented the mean value ± SE of three biological replicates

The symbol ^*^ reflects the color measurment description. It is a standard color measurment notation.

Twenty-eight out of sixty-one *PbrPGs* were expressed during postharvest storage of ‘Housui’ pear; and the transcripts of 14 members could be detected at every stage (Fig. 4b). Based on the distinct expression patterns, they could be divided into five groups: mRNA abundances of genes in Group I, including *PbrPG6*, *10*, *14*, 2*6*, *31*, *35*, 39, *43* and *47,* showed an increased trend throughout storage; the expression of *PbrPG3*, *4*, *17* and *30* in Group II was downregulated; the transcription of members in Group III, such as *PbrPG2*, *46*, *49* and *51*, was inhibited at the early stages before a increment; the expression pattern of genes (*PbrPG1*, *33*, *37*, *48*, *52*, 53, *54*) in Group IV was opposite to that in Group III; on the other hand, the expression of other members in Group V fluctuated during storage. RT-qPCR analysis validated the accuracy of transcriptome data on the expression patterns of *PbrPGs* (Fig. 4b and Additional file 1: Figure S5a).

Correlation analysis among *PbrPGs* mRNAs, PG activity, and firmness were performed, and genes with high correlation coefficients with PG activity as well as firmness (> 70%) were summarized, including *PbrPG4*, *6*, *17*, *26*, *46* and *61* (Fig. 4c). Of these, *PbrPG4*, *6*, *17*, *46* and *61* were cluster with *PpPG15, 21* and *22* (Fig. 1a), which were proposedly involved in the softening process of peach fruit [8]; meanwhile, *PbrPG6* and *26* demonstrated a similar pattern as *PpPG15, 21* and *22* during storage (Fig. 4b and [8]). These results implied that *PbrPG6* might play a key role in fruit softening process.

**Fig. 5:**
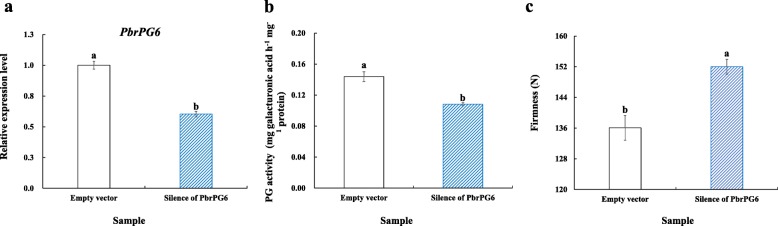
Impact of transient silence of *PbrPG6* expression on PG activity and firmness of pear fruit. **a** Expression profile of *PbrPG6* after transient silence of *PbrPG6* expression. **b** Impact of transient silence of *PbrPG6* expression on PG activity. **c** Impact of transient silence of *PbrPG6* expression on cortex firmness. ‘Housui’ fruit were infiltrated with pTRV2:*PbrPG6* & pTRV1, and then stored at 25 °C before analysis. The unconstructed pTRV2 vector, which was co-injected with pTRV1, was used as control. Data represented the mean ± SD of three biological replicates. The expression level of *PbrPG6* in control fruit was set as 1.0. Vertical bars labeled with different small letters indicated significant difference between samples at *p* < 0.05 level using Duncan’s multiple range test

In order to validate its function, we then constructed the silencing vector for transient transformation of pear fruit. As shown in Fig. 5, a higher firmness was observed for the cortex of transgenic fruit with lower level of *PbrPG6* mRNAs and PG activity, when compared with that of the control.

### Candidate *PbrERFs* regulating the expression of *PbrPG6*

**Fig. 6:**
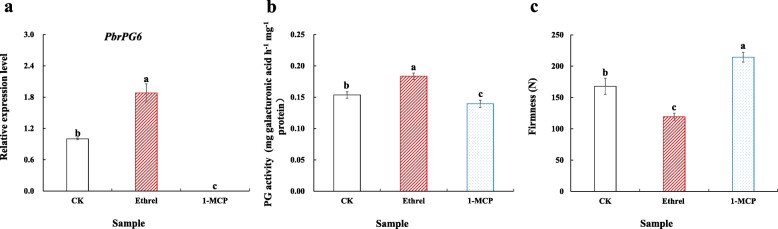
Impact of 1-MCP/ethrel treatment on *PbrPG6* expression, PG activity and cortex firmness of pear. **a** Expression profile of *PbrPG6* after 1-MCP and ethrel treatments. **b** Impact of 1-MCP and ethrel treatments on PG activity. **c** Impact of 1-MCP and ethrel treatments on cortex firmness. ‘Housui’ pears, harvested from an experimental orchard in Nanjing, were divided into three treatments: (1) fumigated with 1.5 μL L^− 1^ 1-MCP for 24 h, (2) dipped in 0.5 mL L^− 1^ ethrel for 5 min, and (3) dipped in 0.5 mL L^− 1^ H_2_O for 5 min (control). After treatments, fruits were packed with plastic bags and stored at 25 °C for 5 d. Data represent the mean ± SE of three biological replicates. The expression level of *PbrPG6* in control fruit was set as 1.0. Vertical bars labeled with different small letters indicated significant difference between samples at *p* < 0.05 level using Duncan’s multiple range test

As shown in Fig. 4c, a positive correlationship was observed between *PbrPG6* mRNAs and ethylene evolution. Besides, an opposite impact on *PbrPG6* expression, PG activity and cortex firmness was observed after 1-MCP and ethrel treatment of pear fruit: 1-MCP fumigation suppressed *PbrPG6* mRNAs and PG activity, resulting in a higher cortex firmness when compared with that of the control; on the other hand, *PbrPG6* expression and PG activity were upregulated by ethrel dipping, and cortex firmness of ethrel-treated fruit was lower than control (Fig. 6). A similar result was observed in a previous study conducted in out unit in 2017 (Additional file 1: Figure S6). Besides, fruit with upregulated expression of *PbrACO1*, which could enhance ethylene evolution [15], demonstrated higher abundances of *PbrPG6* mRNAs as well as lower firmness (Additional file 1: Figure S7). Furthermore, *PbrPG6* contained GCC-box within 2000 bp upstream from the translational starting site (https://www.dna.affrc.go.jp/PLACE/) (Additional file 2: Table S8) [22]*.* These results suggested that the expression of *PbrPG6* might be regulated by ethylene, and PbrERF might participate in this process.

**Fig. 7:**
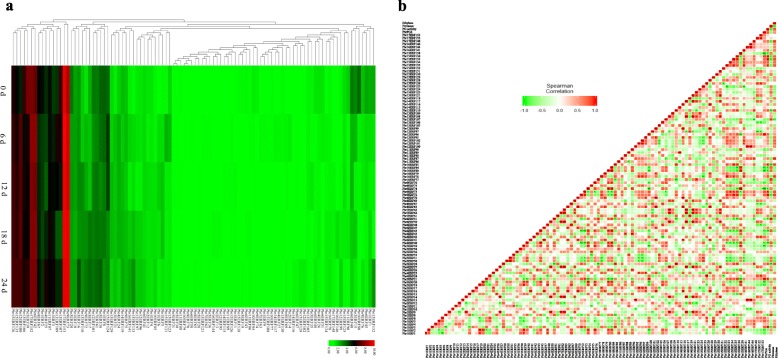
Expression profiles of *PbrERFs* during ‘Housui’ pear storage. **a** Heatmap of the expression profiles of *PbrERFs*. Uniform and defect-free ‘Housui’ pear fruit were selected, randomly divided into several groups, packed with plastic bags, and then stored at 25 °C. Samples were taken every 6 d until decay rate over 20%. Color scale at the top represented log2 transformed (FPKM + 1). Green indicated low level, black indicated a medium level, and red indicated high level. **b** Correlation coefficients among *PbrERF* transcripts, PG activity, ethylene evolution, and *PbrPG6* mRNAs. Pearson correlation between attributes was visualized as a heat map, where negative correlations was represented in green and positive correlations in red

Based on previous report on *ERF* family genes in pear genome [23] as well as transcriptome analysis, 100 out of 155 *PbrERFs* were expressed during ‘Housui’ pear storage with diverse expression patterns (Fig. 7a). RT-qPCR analysis validated the accuracy of transcriptome data on the expression patterns of *PbrERFs* (Additional file 1: Figure S5b). Correlation analysis found that 33 members illustrated high correlation coefficients (> 70%) with *PbrPG6.* Of these, *Pbr1ERF5/6*, *Pbr3ERF21*, *Pbr4ERF24*, *Pbr5ERF28*, *Pbr12ERF100*, *Pbr13ERF110*, *Pbr15ERF126/129/136*, and *Pbr17ERF148* were positively correlated with *PbrPG6*; on the other hand, a negative relationship was observed between *PbrPG6* and other *PbrERFs* (*Pbr1ERF1/2*, *Pbr3ERF15/19/20*, *Pbr4ERF26*, *Pbr5ERF38/39*, *Pbr6ERF40*/*45*/*48*/*51*, *Pbr8ERF67*, *Pbr9ERF74*/*76*, *Pbr10ERF80*/*84*, *Pbr11ERF86*/*90*, *Pbr13ERF106*/*107*, and *Pbr15ERF138*) (Fig. 7b).

Besides, 30 members illustrated high correlation coefficients (> 70%) with ethylene (Fig. 7b). Of these, *Pbr2ERF13*, *Pbr3ERF17*, *Pbr5ERF28*, *Pbr7ERF61*, *Pbr9ERF72*/*73*, *Pbr10ERF78*/*85*, *Pbr11ERF87*, *Pbr12ERF95*–*97*, *Pbr13ERF110*, *Pbr14ERF119*, *Pbr15ERF133*–*136*, *Pbr16ERF144*/*146*, and *Pbr17ERF148* were positively correlated with ethylene*.* On the other hand, a negative relationship was observed between ethylene and other *PbrERFs*, including *Pbr1ERF8*, *Pbr5ERF30*/*31*, *Pbr6ERF51*, *Pbr10ERF77*, *Pbr13ERF105/107*, *Pbr15ERF124*/*125*.

## Discussion

Pear, distributed on six continents with China as the leading producer, is popular among consumers for its unique flavor quality [14, 16]. During ‘Housui’ pear storage, a^*^ value in pericarp, weight loss and decay rate accumulated, while cortex firmness decreased (Table 1). Similar phenomenon was also observed for ‘Yali’ and ‘Huanghua’ pear during storage [14, 19].

Fruit softening, which is mainly due to the alternation in cell wall structure & composition [3], could enhance the sensitivity of pear fruit to mechanical damage and thus shorten their shelf life [2]. Cell wall is consisted of complex polysaccharides, including pectin, cellulose, and hemicellulose [3]. A variety of enzymes, including PG, pectinesterase (PE), β-galactosidase (β-Gal), cellulase and xyloglucan endo-transglycosylase (XET), are proposedly involved in the metabolism of these components [7, 24].

Pectin is a structural polysaccharide of the primary cell wall and middle lamella, playing an important role in cell-to-cell adhesion [7]. PG is the key enzyme involved in its degradation, through cleavage of α-(1 → 4) glycoside bonds [7, 25]. Until recently, *PG* family genes have been characterized from many plants [6, 8, 9, 26]. Besides fruit softening, PG also functions in many developmental processes of plant, such as flower development or abscission zone development [7]. During ‘Huanghua’ pear storage, firmness demonstrated an opposite trend when compared with that of PG activity [19]. A similar phenomenon was observed during ‘Housui’ fruit storage (Fig. 4a and Additional file 2: Table S1). However, our knowledge on *PG* gene family in pear genome as well as their role in fruit softening is still rudimentary.

A total of 61 *PbrPGs* were identified from the pear genome with an uneven chromosomal distribution, which could be divided into six groups (A-F) (Fig. 1a). A similar result was observed in other plants [6–8]. In consistent with the previous report on PGs from in peach, most PbrPGs contained four conserved domains [8]: domains I & II likely composed the catalytic site; domain III was involved in catalytic reaction; on the other hand, domain IV constituted a likely candidate for ionic interaction with carboxylate groups in the substrate [27].

The gene structures and the composition of motifs or *cis*-acting elements were distinct among *PbrPGs*/PbrPGs; members within the same class shared similar gene structure and components (Fig. 1b). Similar result were observed for other gene families in pear [15, 20]. In agreement with the result of Wu et al. [28], most *PbrPGs* were assigned to WGD/segmental duplication, and the purifying selection was the primary driving force for their evolution (Additional file 2: Table S7). Polyploidy through WGD is frequently associated with genome rearrangement, and the evolution of genes is proposedly driven by a variety of factors, including structural complexity, conserved domain, and evolutionary rate [15]. In our study, PbrPGs possessed four highly-conserved domains (Additional file 1: Figure S1), and demonstrated lower Ka/Ks ratios (Additional file 2: Table S7), implying that they were relatively functionally stable over recent years and may function as good targets for dosage balance selection [20]. Besides, their expression profiles in pear were tissue/development-stage/cultivar-specific (Fig. 3 & S3), which was similar to the report on *SlPG* family genes [6].

Twenty-eight *PbrPGs* were expressed during ‘Housui’ pear storage, which could be divided into five groups based on their expression patterns (Fig. 4b). Similarly, 16 out of 45 *PpPG* genes identified from peach genome were transcribed during ripening, with diverse expression profiles [8]; of these, *PpPG15, 21* and *22* might play a critical role in fruit softening [8]. In combination of the results from correlation and phylogenetic analysis, *PbrPG6* might play a key role in the softening process of pear fruit (Fig. 1a & 4c). Silence of *PbrPG6* expression suppressed PG activity and maintained fruit firmness when compared with that of the control (Fig. 5). Previously, Quesada et al. [29] found that antisense of a strawberry *FaPG1* gene inhibited the softening process of the ripened fruit, which might be due to a decrease in pectin solubilization and an enhancement of the amount of pectin covalently bound to the cell wall. Similar phenomenon was also observed by downregulating *PG1* expression in ‘Royal Gala’ apple [30]. These results implied that a higher firmness in *PbrPG6-*silenced fruit might be due to more pectin covalently bound to the cell wall, in comparison with that of the control.

Pear, a climacteric fruit, is characterized by an increase in the respiration rate, which was associated with the accumulation of ethylene, upon initiation of ripening (Trinchero et al., 2004). In consistent with this, both ethylene evolution and respiration rate accumulated with the highest level at 18th d during fruit storage (Additional file 1: Figure S4). Ethylene plays a key role in the quality alternation during climacteric fruit ripening [31]. Mutation of an ethylene receptor, *Never-ripe* (Nr), inhibited the ripening process and quality formation of fruit [11]. In this study, the expression pattern of *PbrPG6* mRNAs and ethylene evolution during pear storage was similar (Fig. 4b & Additional file 1: Figure S4a); meanwhile, the impact of 1-MCP and ethrel treatments on *PbrPG6* mRNAs, PG activity and cortex firmness was opposite (Fig. 6). These results implied that the expression of *PbrPG6* might be under the control of ethylene.

As final response gene in the ethylene signaling pathway, ERF could bind to the promoters of several genes, such as *ACO*, *PME,* and *PG*, regulating ethylene formation and quality alternation [12, 13]; meanwhile, the impact of ERFs on the ripening were diverse [10]. Based on bioinformatic analysis, GCC-box, which ERF could bind to [12], was observed in the promoters of *PbrPG6* (Additional file 2: Table S8), suggesting that PbrERF might regulate the expression of *PbrPG6.* During ‘Housui’ pear storage, 100 *PbrERFs* were expressed, with diverse expression patterns (Fig. 7a); of these, 33 members illustrated relatively high correlation coefficients (> 70%) with *PbrPG6* (Fig. 7b)*.*

## Conclusions

Sixty-one *PbrPGs*, which could be divided into six groups (A-F), were identified from pear genomes with different chromosome locations, gene structures, motifs and *cis*-acting elements. Most genes were derived from WGD/segmental duplication with purifying selection as the main driving force. Their expression profiles in pear were tissue/development-stage/cultivar-specific. During ‘Housui’ pear storage, in association with quality (such as color, weight loss, decay rate, firmness) alternation as well as the accumulation of PG activity, 28 *PbrPG* genes were transcribed, which could be classified into five categories based on different expression patterns. Of these, *PbrPG6* played an important role in fruit softening in combination of bioinformatic analysis & experimental validation. Further study found that its expression might be regulated by ethylene; and several *PbrERFs* might be involved in this process.

## Methods

### Sequence retrial and annotation of PGs from pear

Protein sequences of PGs from peach and *Arabidopsis* genome were downloaded from Genome Database for Rosaceae (GDR) (http://www.rosaceae.org/) [32] and The *Arabidopsis* Information Resource (TAIR) (http://www.arabidopsis.org/) [33], respectively (Additional file 2: Table S9). These sequences were used as queries to perform BLASTP against the pear genome database (http://peargenome.njau.edu.cn/) [28]. Subsequently, the seed alignment file for PG domain (PF00295) which was accessed from Pfam database (http://pfam.sanger.ac.uk/) was used to build a HMM file prior to HMM searches against the local protein database of pear, using HMMER3 [20]. All candidates were then submitted to Pfam or SMART (http://smart.embl-heidelberg.de/) database to verify the presence of conserved domains, and the candidates lacking more than two highly conserved PG domains (domains I (‘SPNTDGI’), II (‘GDDC’), III (‘CGPGHGISIGSLG’), and IV (‘RIK’)) were eliminated [8].

A local BLASTN against the pear EST libraries was conducted to find the records for each putative candidate with a maximum identity > 95%, length > 200 bp, and E-value < 10^− 20^ [20].

The physiological and biochemical parameters of the full-length proteins were calculated, using ProtParam tool (http://web.expasy.org/protparam/) [15]. Signal peptide and subcellular localization of each member were analyzed by SignalP 4.1 (http:/www.cbs.dtu.dk/services/SignalP/) [34] and CELLO v2.5 server (http://cello.life.nctu.edu.tw/) [35], respectively.

### Phylogenetic, gene structure, motif and *cis*-acting element analysis

The phylogenetic tree was constructed by MEGA 7.0.26 software, using the neighbor-joining (NJ) method with a bootstrap analysis of 1000 replicates and the poisson model [36]. Gene structures of *PbrPGs* obtained by alignment of open reading frames (ORFs) with the corresponding genomic sequences along with the Gene Structure Display Server 2.0 (GSDS 2.0) program (http://gsds.cbi.pku.edu.cn/) [15]. Motifs analysis was performed using MEME Suite 5.0.5 [37]; the identified motifs were annotated using SMART database [38]. *Cis*-acting regulatory elements in the 1.5 kb of 5′ regulatory region from the translational start site were identified using PlantCARE database [15]; and the divergence between upstream sequences of each paralogous gene pairs was measured by the GATA program [39], with window size set as seven and the lower cut-off score was 12 bit.

### Chromosomal location, synteny, and Ka/Ks analysis

The chromosome locations of all *PbrPGs* were determined according to genome annotation data, and then plotted using Circos software [40].

A method similar to that used for PGDD (http://chibba.agtec.uga.edu/duplication/) was applied to analyze the syntenic relationship [41]; and the duplicated *PbrPGs* were then categorized into the following types: whole genome duplication (WGD)/segmental, tandem, singleton, proximal and dispersed. MCScanX downstream analysis tools was used to annotate the Ka and Ks substitution rates of the syntenic gene pair; and the KaKs Calculator 2.0 was used to determine Ka and Ks with the Nei-Gojobori (NG) method [42].

### Plant material

Uniform and defect-free pear fruit (*P. pyrifolia* cv. ‘Housui’) harvested from homogeneous trees planted in the experimental orchard of the College of Horticulture at Nanjing Agricultural University were chosen, randomly divided into several groups, packed with plastic bags, and then stored at 25 °C. Samples were taken every 6 d until decay rate over 20%. For the sampling, the cortex from five fruit per replicate was quickly removed with a parer before analysis.

### Color, weight loss, decay rate, firmness, ethylene production and respiration rate determination

Color (pericarp and first layer of the cortex tissue below pericarp), weight loss and decay rate were determined according to the method of Wang et al. [16]. A Minolta CR-400 chromameter (Konica Minolta Sensing, Inc., Osaka, Japan) was used for color analysis.

Cortex firmness was measured by Brookfield texture analyzer (CT3, Middleboro, MA, United States), using a 2-mm stainless cylindrical probe, loading at 0.5 mm s^− 1^ in association with 10 mm distance.

Respiration rate was assayed by YGA2100 CO_2_ analyzer (Yangguangyishida Technology Co. Ltd., Beijing, China) according to the instruction of manufacturer. Ethylene evolution was determined by a GC (Agilent Technologies 7890A) fitted with flame ionization detector (FID) [43].

### Transcriptome sequencing and qRT-PCR analysis

Total RNA was extracted using TRizol Reagents (Invitrogen, Carlsbad, CA) followed by RNase-free DNase treatment (Qiagen, Valencia, CA). RNA-seq and bioinformation analysis were conducted by Biomarker Technologies (Beijing, China). Library construction was carried out using Illumina HiSeqTM 4000 sequencing platform; pear genome database [28] was used as reference genome; FPKM was used to calculate gene expression [44]. Based on previous report, the wild *P. pyrifolia* was a common ancestor for *P. pyrifolia* and *P. bretschneideri* [45].

The primers of *PbrPGs* were designed, using Premier 6.0 (Additional file 2: Table S10). Total RNA was isolated using TRizol Reagents (Invitrogen, USA) followed by RNase-free DNase treatment (Qiagen, USA). Approximately 2 μg of total RNA was used for first-strand cDNA synthesis using TransScript One-Step gDNA Removal and cDNA Synthesis SuperMix (TRANSGEN, China). qRT-PCR was performed according to the method of Wang et al. [15]. *Pyrus* Tubulin was used as the internal control, and the relative expression levels were calculated with the 2^−△△Ct^ method [46].

### PG activity assay

Extraction of crude PG from pear cortex and analysis of PG activity was conducted according to the instruction of manufacturer (PG-1-G, Suzhou Comin Biotechnology Co., Ltd., China). Protein concentration in crude enzyme extract was determined by protein assay kit (SSNP-1-W, Suzhou Comin Biotechnology Co., Ltd., China). The result was expressed as mg galacturonic acid h^− 1^ mg^− 1^ protein.

### Transient silencing of *PbrPG6* expression in pear fruit

About 400 bp fragment at the C-terminal of *PbrPG6* were amplified before insertion into the pTRV2 vector [47]. The constructed vector and pTRV1, which could assist the movement of pTRV2 vector in cell, were transformed into *A. tumefaciens* strain GV3101, respectively; and then combined in a 1:1 ratio before injection into the cortex tissue [48]. The unconstructed pTRV2 vector, which was co-injected with pTRV1, was used as control. The injected fruit were then stored at 25 °C for 5 d before sampling. There were three replicates per treatment with five fruit per replicate.

### Transient transformation of pear fruit

Transient transformation of pear fruit was carried out following Hao et al. [12]‘s method. *PbrACO1* ORFs were amplified (Additional file 2: Table S10), inserted into a modified pCAMBIA 1300 vector, and then transformed into *A. tumefaciens* strain GV3101. After incubation, the suspension was centrifuged, resuspended with the infiltration buffer, and slowly injected into pear cortex before storage at 25 °C. Pear fruit infiltrated with the empty vector were used as control. There were three replicates with five fruit each for each vector.

### Statistical analysis

Data presented were the mean values of at least three biological replicates except for transcriptome analysis of pear fruit during storage (one replicate). SAS version 9.3 (SAS Institute, Gary, NC) was used for data analysis, using analysis of variance (PROC ANOVA) with multi-comparison correction. Mean separation was determined by Duncan’s multiple range test at the 0.05 level. Spearman’s correlation analysis was performed to evaluate the association among attributes, which was visualized as a heatmap.

### Availability of supporting data

The transcriptome clean raw reads data that support the findings of this study have been submitted to NCBI Sequence Read Archive (SRA) under Accession SUB6578158, Bioproject: PRJNA590622. All data generated or analyzed during this study are included in this published article and its supplementary information files.

## Supplementary information

**Additional file 1: Figure S1.** Multiple sequence alignment of PbrPGs**.** The red wireframe parts indicated four typical conserved domains of PbrPGs, which were named as domain I, II, III and IV, respectively. **Figure S2.** Comparative analysis of the 1.5 kb upstream of paralogous gene pairs. Divergence between upstream sequences of each paralogous gene pairs was measured by the GATA program (Nix and Eisen, 2005), with window size set as seven and lower cutoff score 12 bit. Solid dark lines connect similar regions and red broken lines connect matched regions in reversed orientation. **Figure S3.** Expression profiles of *PbrPGs* during development of different pear fruit. ‘Housui’ (a), ‘Kuerlexiangli’ (b), ‘Nanguo’ (c), ‘Starkrimson’ (d), ‘Yali’ (e) fruit were harvested from a commercial field at five developmental stages, including fruit-setting stage (period 1), physiological fruit dropping stage (period 2), fruit rapid enlargement stage (period 3), a month after fruit enlargement stage (period 4), and commercially mature stage (period 5). Data adapted from Zhang et al. (2015). Color scale at the top represented log2 transformed (FPKM + 1). Green indicated low level, black indicated a medium level, and red indicated high level. **Figure S4.** Dynamic changes of ethylene evolution and respiration rate during ‘Housui’ pear storage. Uniform and defect-free ‘Housui’ pear fruit were selected, randomly divided into several groups, packed with plastic bags, and then stored at 25 °C. Samples were taken every 6 d until decay rate over 20%. Data represented the mean value ± SE of three biological replicates. Different small letters with the same treatment mean significant difference among samples (*p* < 0.05). **Figure S5.** qRT-PCR validation of the expression patterns of genes based on transcriptome analysis. Uniform and defect-free ‘Housui’ pear fruit were selected, randomly divided into several groups, packed with plastic bags, and then stored at 25 °C. Samples were taken every 6 d until decay rate over 20%. Data represented the mean ± SE of three biological replicates for qRT-PCR analysis. The expression level of *PbrPG1* and *Pbr5ERF39* at 0th d was set as 1.0. Different small letters with the same treatment mean significant difference among samples (*p* < 0.05). **Figure S6.** Impact of 1-MCP and ethrel treatments on cortex firmness during pear storage. ‘Housui’ pears were harvested from an experimental orchard in Nanjing in 2017, and then divided into three treatments: (1) fumigated with 1.5 μL L^− 1^ 1-MCP for 24 h, (2) dipped in 0.5 mL L^− 1^ ethrel for 5 min, and (3) dipped in 0.5 mL L^− 1^ H_2_O for 5 min (control). After treatments, fruits were packed with plastic bags and stored at 25 °C. Samples were taken every 6 d. Data represent the mean ± SE of three biological replicates. Different lowercase letters with the same treatment mean significant difference among samples, and different capital letters in the same sampling data mean significance among treatments (*p* < 0.05). **Figure S7.** Impact of transient overexpression of *PbrACO1* on *PbrPG6* mRNAs and firmness of pear fruit. (a) Expression profile of *PbrACO1* in samples. (b) Impact of transient on *PbrPG6* mRNAs. (c) Impact of overexpression of *PbrACO1* on cortex firmness. ‘Housui’ fruit infiltrated with the empty vector was used as control. Data represented the mean ± SE of three biological replicates. Different lowercase letters meant significance between samples (*p* < 0.05). The expression level of *PbrACO1/PbrPG6* in control fruit was set as 1.0. Vertical bars labeled with different small letters indicated significant difference between samples at *p* < 0.05 level using Duncan’s multiple range test.

**Additional file 2: Table S1.** Information on 61 *PbrPGs* from pear genome. **Table S2.** ESTs for putative *PbrPGs.* **Table S3.** Gene features of *PG* family genes from pear. **Table S4.** *Cis*-acting regulatory elements identified in the promoters of *PbrPGs.* **Table S5.** Motif sequences identified by MEME tools in PbrPGs. **Table S6.** Duplication types of *PbrPG* genes in pear genome. **Table S7.** Ka/Ks ratios of paralogous genes in *PbrPG* gene family. **Table S8.** Promoters analysis of *PbrPG6* using PLACE Web Signal Scan. **Table S9.** Information of *PG* genes from *Arabidopsis* and peach. **Table S10.** Primers used in this study. (XLS 667 kb)

## Data Availability

Not applicable.

## Abbreviations

1-MCP: 1-Methylcyclopropene
ACO: Acid oxidase
DAFB: Days after full bloom
EMSA: Electrophoretic mobility shift assay
ERF: Ethylene response factors
ESTs: Expressed sequence tags
FID: Flame ionization detector
FPKM: Fragments per kilobase million
GDR: Genome database for Rosaceae
GRAVY: Grand average of hydropathicity
GSDS: Gene structure display server
HMM: Hidden markov model
MEME: Multiple EM for motif elicitation
MYA: Million years ago
NG: Nei-Gojobori
NJ: Neighbor-joining
Nr: Never-ripe
ORFs: Open reading frames
PE: Pectinesterase
PG: Polygalacturonase
PGDD: Plant genome duplication database
PI: Isoelectric point
PME: Pectin methylesterase
TAIR: The arabidopsis information resource
WGD: Whole genome duplication
XET: Xyloglucan endo-transglycosylase
β-Gal: β-galactosidase
