# Supplementary material for: Genome-wide analysis of polygalacturonase gene family from pear genome and identification of the member involved in pear softening
Source: BMC Plant Biol. 2019 Dec 27;19:587. doi: 10.1186/s12870-019-2168-1 (PMC6935220; doi:10.1186/s12870-019-2168-1)
Supplement: Supplementary file 1 — Additional file 1: Figure S1. Multiple sequence alignment of PbrPGs. The red wireframe parts indicated four typical conserved domains of PbrPGs, which were named as domain I, II, III and IV, respectively. Figure S2. Comparative analysis of the 1.5 kb upstream of paralogous gene pairs. Divergence between upstream sequences of each paralogous gene pairs was measured by the GATA program (Nix and Eisen, 2005), with window size set as seven and lower cutoff score 12 bit. Solid dark lines connect similar regions and red broken lines connect matched regions in reversed orientation. Figure S3. Expression profiles of PbrPGs during development of different pear fruit. ‘Housui’ (a), ‘Kuerlexiangli’ (b), ‘Nanguo’ (c), ‘Starkrimson’ (d), ‘Yali’ (e) fruit were harvested from a commercial field at five developmental stages, including fruit-setting stage (period 1), physiological fruit dropping stage (period 2), fruit rapid enlargement stage (period 3), a month after fruit enlargement stage (period 4), and commercially mature stage (period 5). Data adapted from Zhang et al. (2015). Color scale at the top represented log2 transformed (FPKM + 1). Green indicated low level, black indicated a medium level, and red indicated high level. Figure S4. Dynamic changes of ethylene evolution and respiration rate during ‘Housui’ pear storage. Uniform and defect-free ‘Housui’ pear fruit were selected, randomly divided into several groups, packed with plastic bags, and then stored at 25 °C. Samples were taken every 6 d until decay rate over 20%. Data represented the mean value ± SE of three biological replicates. Different small letters with the same treatment mean significant difference among samples (p < 0.05). Figure S5. qRT-PCR validation of the expression patterns of genes based on transcriptome analysis. Uniform and defect-free ‘Housui’ pear fruit were selected, randomly divided into several groups, packed with plastic bags, and then stored at 25 °C. Samples were taken every 6 d until decay [file 12870_2019_2168_MOESM1_ESM.docx]

**Additional Files 1**

**Fig. S1. Multiple sequence alignment of PbrPGs.** The red wireframe parts indicated four typical conserved domains of PbrPGs, which were named as domain I, II, III and IV, respectively.

**Fig. S2. Comparative analysis of the 1.5 kb upstream of paralogous gene pairs.** Divergence between upstream sequences of each paralogous gene pairs was measured by the GATA program (Nix and Eisen, 2005), with window size set as seven and lower cutoff score 12 bit. Solid dark lines connect similar regions and red broken lines connect matched regions in reversed orientation.

**Fig. S3. Expression profiles of *PbrPGs* during development of different pear fruit.** ‘Housui’ (a), ‘Kuerlexiangli’ (b), ‘Nanguo’ (c), ‘Starkrimson’ (d), ‘Yali’ (e) fruit were harvested from a commercial field at five developmental stages, including fruit-setting stage (period 1), physiological fruit dropping stage (period 2), fruit rapid enlargement stage (period 3), a month after fruit enlargement stage (period 4), and commercially mature stage (period 5). Data adapted from Zhang et al. (2015). Color scale at the top represented log2 transformed (FPKM + 1). Green indicated low level, black indicated a medium level, and red indicated high level.

**Fig. S4. Dynamic changes of ethylene evolution and respiration rate during ‘Housui’ pear storage.** Uniform and defect-free ‘Housui’ pear fruit were selected, randomly divided into several groups, packed with plastic bags, and then stored at 25℃. Samples were taken every 6 d until decay rate over 20%. Data represented the mean value ± SE of three biological replicates. Different small letters with the same treatment mean significant difference among samples (*p* < 0.05).

**Fig. S5. qRT-PCR validation of the expression patterns of genes based on transcriptome analysis.** Uniform and defect-free ‘Housui’ pear fruit were selected, randomly divided into several groups, packed with plastic bags, and then stored at 25℃. Samples were taken every 6 d until decay rate over 20%. Data represented the mean ± SE of three biological replicates for qRT-PCR analysis. The expression level of *PbrPG1* and *Pbr5ERF39* at 0^th^ d was set as 1.0. Different small letters with the same treatment mean significant difference among samples (*p* < 0.05).

**Fig. S6. Impact of 1-MCP and ethrel treatments on cortex firmness during pear storage.** ‘Housui’ pears were harvested from an experimental orchard in Nanjing in 2017, and then divided into three treatments: (1) fumigated with 1.5 μL L^−1^ 1-MCP for 24 h, (2) dipped in 0.5 mL L^−1^ ethrel for 5 min, and (3) dipped in 0.5 mL L^−1^ H_2_O for 5 min (control). After treatments, fruits were packed with plastic bags and stored at 25 ℃. Samples were taken every 6 d. Data represent the mean ± SE of three biological replicates. Different lowercase letters with the same treatment mean significant difference among samples, and different capital letters in the same sampling data mean significance among treatments (*p* < 0.05).

**Fig. S7. Impact of transient overexpression of *PbrACO1* on *PbrPG6* mRNAs and firmness of pear fruit.** (a) Expression profile of *PbrACO1* in samples. (b) Impact of transient on *PbrPG6* mRNAs. (c) Impact of overexpression of *PbrACO1* on cortex firmness. ‘Housui’ fruit infiltrated with the empty vector was used as control. Data represented the mean ± SE of three biological replicates. Different lowercase letters meant significance between samples (*p* < 0.05). The expression level of *PbrACO1/PbrPG6* in control fruit was set as 1.0. Vertical bars labeled with different small letters indicated significant difference between samples at *p* < 0.05 level using Duncan’s multiple range test.
